# Ecological and sanitary impacts of bacterial communities associated to biological invasions in African commensal rodent communities

**DOI:** 10.1038/s41598-017-14880-1

**Published:** 2017-11-03

**Authors:** Christophe Diagne, Maxime Galan, Lucie Tamisier, Jonathan d’Ambrosio, Ambroise Dalecky, Khalilou Bâ, Mamadou Kane, Youssoupha Niang, Mamoudou Diallo, Aliou Sow, Philippe Gauthier, Caroline Tatard, Anne Loiseau, Sylvain Piry, Mbacké Sembène, Jean-François Cosson, Nathalie Charbonnel, Carine Brouat

**Affiliations:** 1 CBGP, IRD, CIRAD, INRA, Montpellier SupAgro, Univ. Montpellier, Montpellier, France; 2 BIOPASS (IRD-CBGP, ISRA, UCAD), Campus de Bel-Air, BP 1386, CP, 18524 Dakar, Senegal; 3 Département de Biologie Animale, Faculté des Sciences et Techniques, Université Cheikh Anta Diop (UCAD), BP 5005 Fann, Dakar, Senegal; 4 CBGP, INRA, CIRAD, IRD, Montpellier SupAgro, Univ. Montpellier, Montpellier, France; 5 IRD, Aix Marseille Univ, LPED, Marseille, France; 6 UMR BIPAR, INRA, ANSES, Ecole Nationale Vétérinaire d’Alfort, Université Paris-Est, Maisons-Alfort, 94700 France

## Abstract

Changes in host-parasite ecological interactions during biological invasion events may affect both the outcome of invasions and the dynamics of exotic and/or endemic infections. We tested these hypotheses, by investigating ongoing house mouse (*Mus musculus domesticus*) and black rat (*Rattus rattus*) invasions in Senegal (West Africa). We used a 16S gene rRNA amplicon sequencing approach to study potentially zoonotic bacterial communities in invasive and native rodents sampled along two well-defined independent invasion routes. We found that individual host factors (body mass and sex) were important drivers of these bacterial infections in rodents. We observed that the bacterial communities varied along invasion routes and differed between invasive and native rodents, with native rodents displaying higher overall bacterial diversity than invasive rodents. Differences in prevalence levels for some bacterial Operational Taxonomic Units (OTUs) provided support for ecological processes connecting parasitism and invasion success. Finally, our results indicated that rodent invasions may lead to the introduction of exotic bacterial genera and/or to changes in the prevalence of endemic ones. This study illustrates the difficulty of predicting the relationship between biodiversity and disease risks, and advocate for public health prevention strategies based on global pathogen surveillance followed by accurate characterization of potential zoonotic agents.

## Introduction

There is a close link between biological invasions (the establishment and spread of a non-native species in a new range) and parasites (term used here to encompass both macroparasites and microparasites), with potential two-way feedback^1^. Parasites may alter the outcome of biological invasions by affecting host fitness and/or changing the strength of interactions between introduced and native hosts^2^. As such, native parasites may prevent new host invasions if they infect the introduced hosts, with detrimental (direct and/or indirect) effects on their fitness^3^. Native parasite pressure in the resident hosts may also be reduced by the introduction of new hosts, through dilution or density effects^4^, indirectly increasing the ability of native hosts to compete against invaders. Conversely, parasites may favour invasion success if the introduction of the new host results in the loss of some or all of the exotic host’s parasites (enemy release hypothesis^5^). Indeed, small numbers of individuals are generally introduced, and they may not carry the complete range of parasites found at the source site; furthermore, introduced parasites may fail to thrive in the new environment^6^. Such decrease in the parasite population would be predicted to enhance the fitness and competitive ability of the introduced species in the new range. Moreover, parasites common to both native and introduced hosts may also favour invasion success, through adverse effects on the fitness and/or survival of native competitors. Invaders may introduce and transmit new parasites to native hosts (“novel weapon” hypothesis^3^) or they may acquire and better amplify endemic parasites, resulting in a higher prevalence/abundance in native hosts than expected in the absence of the new hosts (spillback hypothesis^7^).

Invasion-related changes in host community structure and diversity may also influence the dynamics of diseases potentially affecting wildlife, livestock and/or humans^8^. Disease emergence events associated with non-native pathogens imported by animal invaders have already been observed^1^. Despite growing interest in these issues in recent decades, much remains unknown about the relationships between parasite ecology, disease dynamics and the invasion process^8^. In particular, few studies have examined the concomitant effects of native and invasive host communities on infectious disease risks in invaded ecosystems.

In this study, we examined ecological interactions between native and invasive host rodents and their bacterial communities in the context of well-studied ongoing invasions in Senegal (West Africa). We focused on two major invasive species (Global Invasive Species Database - http://www.issg.org/database/): the house mouse *Mus musculus domesticus* ^9,10^; and the black rat *Rattus rattus* ^11^. Historical records and molecular analyses have shown that these rodents were first brought to sea ports in Senegal by Europeans during the colonial period, and that they remained in coastal villages and towns until the beginning of the 20^th^ century. Both taxa have spread further inland over the last century, via well-defined invasion routes, due to the increase in human activities and the improvement of transport infrastructures in mainland Senegal. The house mouse is now present in most of northern and central Senegal, whereas the black rat is distributed throughout the southern part of the country. Such spatial distribution pattern is likely to result from a combination of historical contingencies, interspecific interactions and environmental effects^9^. Along their invasion routes, these invasive species have colonised human settlements (towns or villages), from which they have progressively evicted the commensal native rodent species, the Guinea multimammate mouse *Mastomys erythroleucus* and the Natal multimammate mouse *Mastomys natalensis*. The invasive and native rodent species coexist at the invasion fronts^9,12^.

Parasites have been reported to play an important role in successful colonisation by house mice and black rats, particularly in insular ecosystems^13,14^. More recently, Diagne *et al*.^15^ have revealed the existence of an association between the loss of gastrointestinal helminths and range expansion for these invasive rodents in Senegal. The invasive and African rodent species studied here are known to carry a large array of zoonotic agents^16^. Changes in the risks of zoonotic diseases for humans along the mouse and rat invasion routes therefore seem likely.

We focused here on the zoonotic bacterial operational taxonomic units (OTUs) detected in rodents by the 16S gene rRNA amplicon sequencing approach described in a previous study^17^. Zoonotic OTUs were defined as OTUs that include at least one bacterial species known or suspected to be pathogenic in mammals and can be transmitted to humans, then leading to critical illnesses^16–18^. We admit OTUs may encompass several bacterial species, some being zoonotic and other not or of unknown status, however detailed phylogenetic analyses for the OTUs selected showed that it was unlikely for the OTUs we considered^17^. We first analysed the relationships between rodent-bacteria interactions and ongoing invasions, by testing for the patterns expected as a result of the ecological processes described above: (i) a decrease in bacterial OTU richness and prevalence in invasive rodents at invasion fronts compared with their original coastal sites, as expected under the enemy release hypothesis; (ii) changes in bacterial OTU richness and prevalence in native rodents from invaded areas relative to those from non-invaded areas: under the novel weapon/spillback hypotheses, an increase would be expected for bacterial OTUs common to native and invasive rodents at invasion fronts, whereas, under the dilution/density effect hypothesis, a decrease would be expected. Finally, we discuss the importance of these variations in bacterial communities along both rodent invasion routes in terms of their implications for public health.

## Results

**Figure 1:**
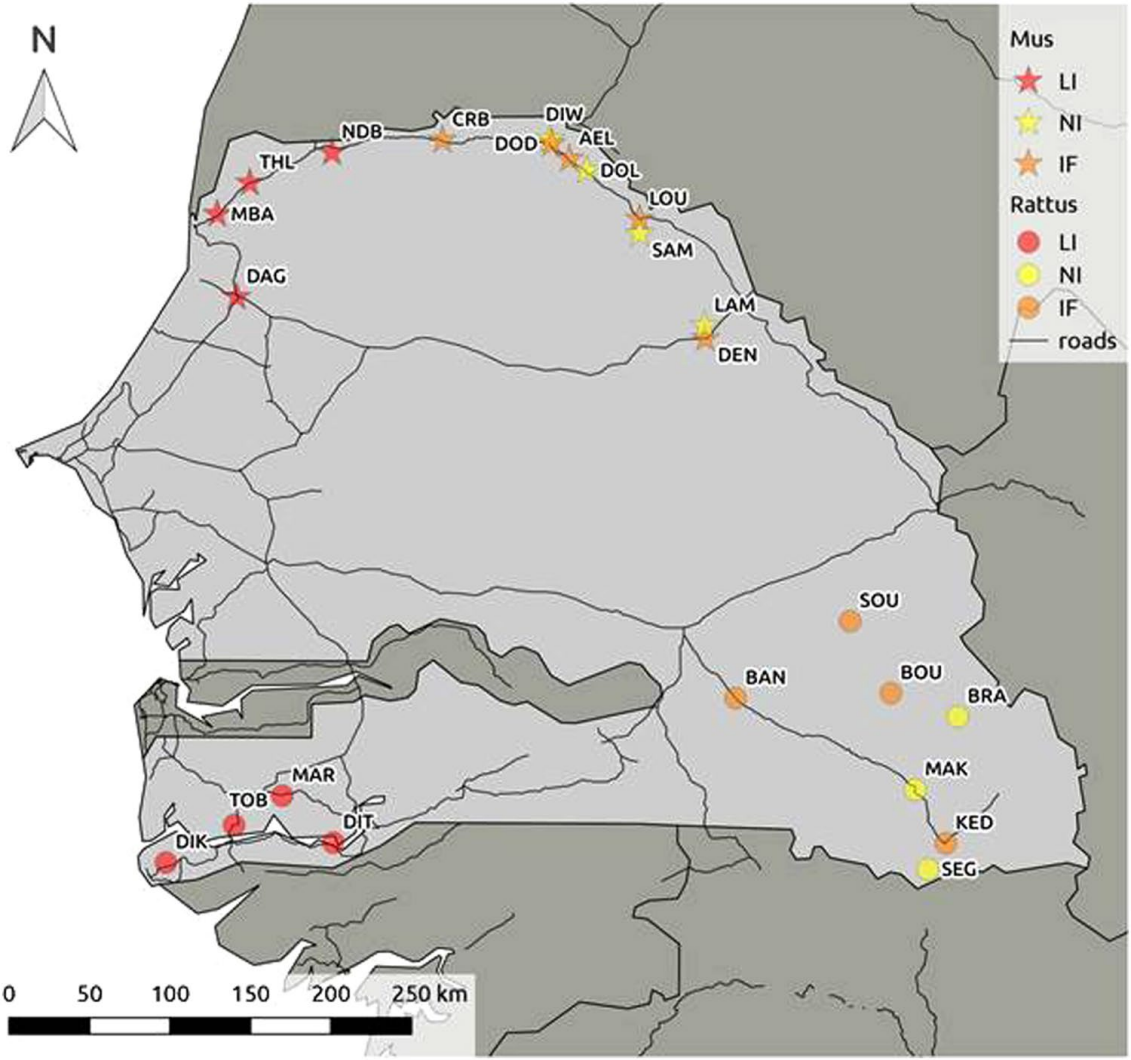
Rodent sampling sites on the mouse (*Mus musculus domesticus*) (stars) and rat (*Rattus rattus*) (circles) invasion routes in Senegal (West Africa). Colour code: red for sites of long-established invasion (LI: rodent communities dominated by invasive species); orange for recently invaded sites or the invasion front (IF: sympatric areas for invasive rodents and native *Mastomys erythroleucus*, or *Mastomys natalensis* at KED); yellow for non-invaded sites (NI: only native rodents, *Ma*. *erythroleucus* in the north, *Ma*. *natalensis* in the south). Correspondence between site codes and names are detailed in Tables 1 and 2. The map was created using QGIS software v. 2.18.7 (http://www.qgis.org/fr/site/).

We sampled a total of 985 rodents from 24 sites (Fig. 1). Trapping sessions showed variations in rodent communities along both invasion routes, from the western coastal sites of long-established invasions to inland invasion fronts and non-invaded sites (Supplementary Table S1; Fig. 1). For convenience, we further used the terms ‘mouse invasion route’ and ‘rat invasion route’ to refer, respectively, to the house mouse and black rat invasion routes studied here.

Along the mouse invasion route (Supplementary Table S1A), only invasive *M*. *m*. *domesticus* was captured at sites of long-established invasion. At the invasion front, native *Ma*. *erythroleucus* co-occurred with *M*. *m*. *domesticus*. As expected, only native rodents were sampled at non-invaded sites.

Along the rat invasion route (Supplementary Table S1B), native rodent communities belonged to two sibling species, *Ma*. *erythroleucus* and *Ma*. *natalensis*. Sites of long-established invasion were highly dominated by invasive *R*. *rattus*. At the invasion front, *R*. *rattus* co-occurred with *Ma*. *erythroleucus* at three of the four sites, and with *Ma*. *natalensis* at the fourth and southernmost site (Kedougou). Only native rodents were sampled at non-invaded sites, mostly belonging to the species *Ma*. *natalensis*. A few *Ma*. *erythroleucus* individuals were sampled at one non-invaded site (Bransan) and at sites of long-established invasion. These individuals probably came inside dwellings from the surrounding fields^12^ and are suspected to be not long-term residents in houses from these sites. We did not include them in subsequent statistical analyses due to their small sample sizes.

Bacteria were successfully screened in 679 rodents (Tables 1 and 2), mainly adults. Twelve zoonotic OTUs were described^17^: *Bartonella*, *Borrelia*, *Ehrlichia*, *Mycoplasma_1*, *Mycoplasma_*2, *Mycoplasma_3*, *Mycoplasma_4*, *Mycoplasma_5*, *Mycoplasma_6*, *Orientia*, *Rickettsia* and *Streptobacillus*.

**Table 1:** Prevalence in % [with 95% confidence intervals calculated with Sterne’s exact method] of bacterial OTUs detected in *Mus musculus domesticus* and *Mastomys erythroleucus* for each site sampled on the mouse invasion route.

Host species	Sites	*Bartonella*	*Borrelia*	*Ehrlichia*	*Mycoplasma_1*	*Mycoplasma_3*	*Orientia*
*M. m. domesticus*	DAG (LI)				29.2 [13.9–50.0]		54.2 [34–73.3]
	MBA (LI)		3.8 [0.2–18.8]	7.7 [4.0–25.0]	11.5 [3.2–30.4]		
	NDB (LI)	4.8 [0.3–23.3]					38.1 [19.7–60.0]
	THL (LI)				21.1 [7.5–44.6]	11.5 [3.2–30.4]	11.5 [3.2–30.4]
	AEL (IF)		4.0 [0.2–19.6]		48.0 [29.6–68.3]		28.0 [13.4–48.0]
	CRB (IF)		3.7 [0.2–18.1]		3.7 [0.2–18.1]		11.0 [3.1–29.2]
	DEN (IF)			33.3 [14.2–60.3]			6.7 [0.4–30.2]
	DOD (IF)				10.0 [1.8–32.0]		40.0 [20.9–63.0]
	LOU (IF)		20.0 [8.2–40.0]	20.0 [8.2–40.0]			4.0 [0.1–26.5]
*Ma. erythroleucus*	AEL (IF)			20.0 [7.1–42.4]	5.0 [0.2–24.4]	45.0 [24.2–68]	
	DEN (IF)		13.6 [3.28- 33.8]	9.1 [1.6–29.1]	40.9 [22.2–61.7]	77.3 [54.7–90.6]	
	DOD (IF)			25.0 [10.4–47.5]		5.0 [0.3–24.4]	
	LOU (IF)	5.0 [0.3–24.4]	25.0 [10.4–47.5]	55.0 [32.0–75.6]			
	DIW (NI)	38.5 [16.6–65.8]		30.8 [11.3–58.6]	53.8 [26.1–77.6]		
	DOL (NI)		10.0 [1.8–32.0]	15.0 [4.2–37.2]		30.0 [14.0–52.5]	
	LAM (NI)	9.5 [1.7–30.5]	33.3 [15.9–55.5]	33.3 [15.9–55.5]	28.6 [13.3–50.6]	52.4 [30.5–72.4]	
	SAM (NI)		22.2 [4.11–55.8]	33.3 [9.8–67.7]			

LI: site of long-established invasion (only invasive host species); IF: invasion front (invasive + native host species); NI: non-invaded sites (only native host species). AEL: Aere Lao; CRB: Croisement Boube; DAG: Dagathie; DEN: Dendoudi; DIW: Diomandou Walo; DOD: Dodel; DOL: Doumnga Lao; LAM: Lambago; LOU: Lougue; MBA: Mbakhana; NDB: Ndombo; THW: Thiewle; THL: Thilene.

**Table 2:** Prevalence in % [with 95% confidence intervals calculated with Sterne’s exact method] of bacterial OTUs detected in *Rattus rattus*, *Mastomys erythroleucus* and *Mastomys natalensis* for each sampling site from the rat invasion route.

Host species	Sites	*Bartonella*	*Borrelia*	*Ehrlichia*	*Mycop-lasma_1*	*Mycop-lasma_2*	*Mycop-lasma_3*	*Mycop-lasma_4*	*Mycop-lasma_5*	*Mycop-lasma_6*	*Orientia*	*Rickettsia*	*Streptobacillus*
*R. rattus*	DIK (LI)					20.0 [8.2–39.8]							4.0 [0.2–19.6]
	DIT (LI)	11.1 [3.1–29.2]				51.9 [33.1–70.8]		33.3 [18.1–53.8]					11.1 [3.1–29.2]
	MAR (LI)					72 [52.0–86.6]		24.0 [11.0–43.9]					
	TOB (LI)	4.8 [0.3–23.3]				81.0 [59.7–93.2]						4.8 [0.3–23.3]	
	BAN (IF)					61.9 [40.3–80.3]		9.5 [1.7–30.5]					
	BOU (IF)	3.6 [0.2–17.5]			3.6 [0.2–17.5]	25.0 [12.0–44.6]	3.6 [0.2–17.5]						
	KED (IF)	4.8 [0.3–23.3]											4.8 [0.3–23.3]
	SOU (IF)		27.3 [12.6–50.0]	36.4 [18.7–58.2]		72.7 [50.0–87.4]		4.5 [0.1–22.9]					
*Ma erythroleucus*	BAN (IF)	53.8 [26.1–77.6]					53.8 [26.1–77.6]						23.1 [6.6–52.0]
	BOU (IF)	90.5 [69.5–98.3]			4.8 [0.3–23.3]		71.4 [49.4–86.8]						14.3 [4.0–35.4]
	SOU (IF)	8.3 [0.4–37.0]	16.7 [3.1–45.7]	41.8 [18.1–70.6]	16.7 [3.1–45.7]		58.3 [29.4–81.9]		8.3 [0.4–37.0]	8.3 [0.4–37.0]			16.7 [3.1–45.7]
*Ma natalensis*	KED (IF)	59.1 [38.3–77.8]			59.1 [38.3–77.8]		13.6 [3.8–33.8]						4.5 [0.2–22.2]
	BRA (NI)	73.9 [52.2–88.0]			43.5 [24.7–64.0]		8.7 [1.6–27.8]		8.7 [1.6–27.8]	21.7 [9.0–43.3]	4.3 [0.2–21.3]		
	MAK (NI)	83.3 [62.8–94.1]			41.7 [23.4–62.8]		58.3 [37.3–76.6]			20.8 [8.6–41.5]	4.2 [0.2–20.4]		
	SEG (NI)	95.8 [79.6–99.8]			37.5 [20.4–58.5]		87.5 [69.0–96.5]		8.3 [1.5–26.7]	12.5 [3.5–31.0]			

LI: site of long-established invasion; IF: invasion front (invasive + native host species); NI: non-invaded sites (only native host species). BAN: Badi Nieriko; BOU: Boutougoufara; BRA: Bransan; DIT: Diattacounda; DIK: Diakene-Wolof; KED: Kedougou; MAK: Mako; MAR: Marsassoum; SEG: Segou; SOU: Soutouta; TOB: Tobor.

### Distribution of bacterial OTUs along invasion routes

Along the mouse invasion route, six zoonotic OTUs were found (Table 1): *Bartonella*, *Borrelia*, *Ehrlichia*, *Mycoplasma_1*, *Mycoplasma_3* and *Orientia*. *Orientia* was found exclusively in *M*. *m*. *domesticus*. All other OTUs were found in both *Ma*. *erythroleucus* and *M*. *m*. *domesticus*. *Mycoplasma_1*, *Ehrlichia* and *Borrelia* were found at sites of all invasion-related categories (sites of long-established invasion, invasion front and non-invaded sites). *Bartonella* and *Mycoplasma_3* were detected in *M*. *m*. *domesticus* at only one site of long-established invasion, whereas these OTUs were found in *Ma*. *erythroleucus* at both the invasion front and non-invaded sites.

Along the rat invasion route, 12 zoonotic OTUs were recorded (Table 2): *Bartonella*, *Borrelia*, *Ehrlichia*, *Mycoplasma_1*, *Mycoplasma_2*, *Mycoplasma_3*, *Mycoplasma_4*, *Mycoplasma_5*, *Mycoplasma_6*, *Orientia*, *Rickettsia* and *Streptobacillus*. Six OTUs were host-specific: *Mycoplasma_2*, *Mycoplasma_4*, and *Rickettsia* in *R. rattus*, *Orientia* in *Ma. natalensis*, *Mycoplasma_5* and *Mycoplasma_6* in *Mastomys* spp. The six OTUs detected in both native and invasive rodents had different distribution patterns. Bartonella was found at sites of all invasion-related categories. *Borrelia* and *Ehrlichia* were restricted to one site at the invasion front. *Mycoplasma_1* and *Mycoplasma_3* were detected in *R. rattus* at one site at the invasion front, and in *Mastomys* spp. at both the invasion front and non-invaded sites.

**Figure 2:**
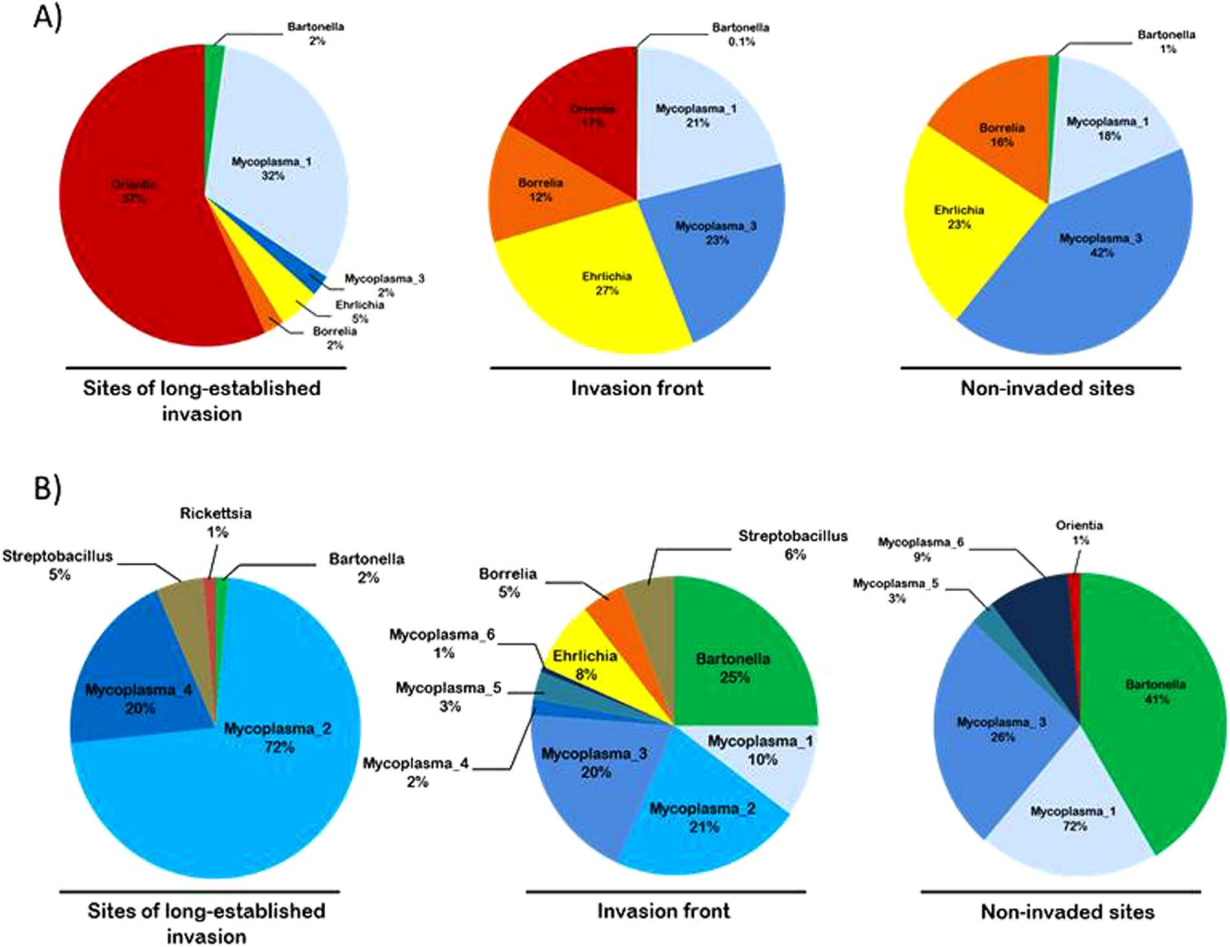
Relative prevalence of the OTUs detected along the mouse (**A**) and rat (**B**) invasion routes highlighting the difference in OTU composition - and then in disease risk - between the three categories of sites sampled.

**Figure 3:**
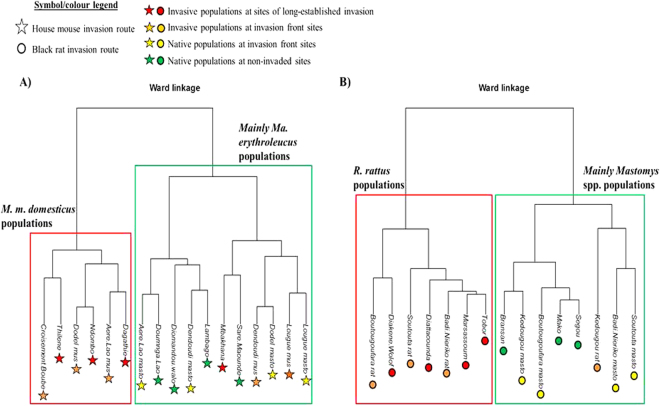
Bray-Curtis (BC) dissimilarity index-based Ward’s hierarchical clustering of zoonotic OTU communities in the rodent host populations sampled along the mouse (**A**) and rat (**B**) invasion route. The graphs indicate that the bacterial communities are mainly clustered by the host species as confirmed by permutational multivariate analyses of variance performed on BC index-based matrices (mouse invasion route: total variance explained *R* ^*2*^ = 0.29; *F* = 5.77, *p* = *0*.*00*2; rat invasion route: *R* ^2^ = 0.52; *F* = 14.35, *p* < *0*.*001*) but not by the specific invasion–related category of sampling sites. The symbols used for host populations are stars and circles for the mouse and rat invasion routes, respectively. Colour legend: red: *Mus musculus domesticus*/*Rattus rattus* populations at sites of long-established invasion; orange: *M*. *m*. *domesticus*/*R*. *rattus* populations at sites at invasion fronts; yellow stars: *Mastomys* spp. populations at sites at invasion fronts; green stars: *Mastomys* spp. populations at non-invaded sites. At invasion front sites where both native and invasive species coexisted, “mus”, “rat” or “masto” has been added after the site name to distinguish between *M*. *m*. *domesticus*, *R*. *rattus* and *Mastomys* spp. populations, respectively. *Mus* (Mbakhana, Dendoudi and Lougue sites) and *Rattus* (Kedougou site) populations that appears in the green boxes (mainly native *Mastomys* populations) of the graphs corresponded to low or null prevalence of the OTUs (*Orientia* for the house mouse, *Mycoplasma*_*2* for the black rat) that dominated bacterial communities of invasive rodents in other localities.

For both invasion routes, our data highlighted differences in zoonotic bacterial communities between the three categories of sites in terms of OTU composition as well as relative prevalence (Fig. 2). Hierarchical clustering and permutational multivariate analysis of variance (permanova) on Bray-Curtis (BC) dissimilarity index-based matrices revealed that the bacterial OTU communities differed significantly between invasive and native host species (mouse invasion route: BC total variance explained *R* ^*2*^ = 0.29; *F* = 5.77, *p* = *0*.*00*2, Fig. 3A; rat invasion route: BC *R* ^2^ = 0.52; *F* = 14.35, *p* < *0*.*001*, Fig. 3B). In contrast, bacterial communities did not differed significantly between invasion-related categories of sites at intraspecific level, for invasive rodents (mouse invasion route: BC *R* ^*2*^ = 0.08; *F* = 0.65, *p* = *0*.*608*; rat invasion route: BC *R* ^2^ = 0.07; *F* = 0.46, *p* = *0*.*884*) as well as for native ones (mouse invasion route: BC *R* ^2^ = 0.06; *F* = 0.39, *p* = *0*.*799*; rat invasion route: BC *R* ^2^ = 0.34; *F* = 2.58, *p* = *0*.*114*). This suggests that the spatial differences in bacterial OTU communities could be the result of changes in rodent host composition along the invasion routes. The same analyses performed with the presence/absence data-based Jaccard dissimilarity index showed similar bacterial community structure patterns (data not shown).

### Factors shaping bacterial communities

#### Bacterial OTU richness

**Figure 4:**
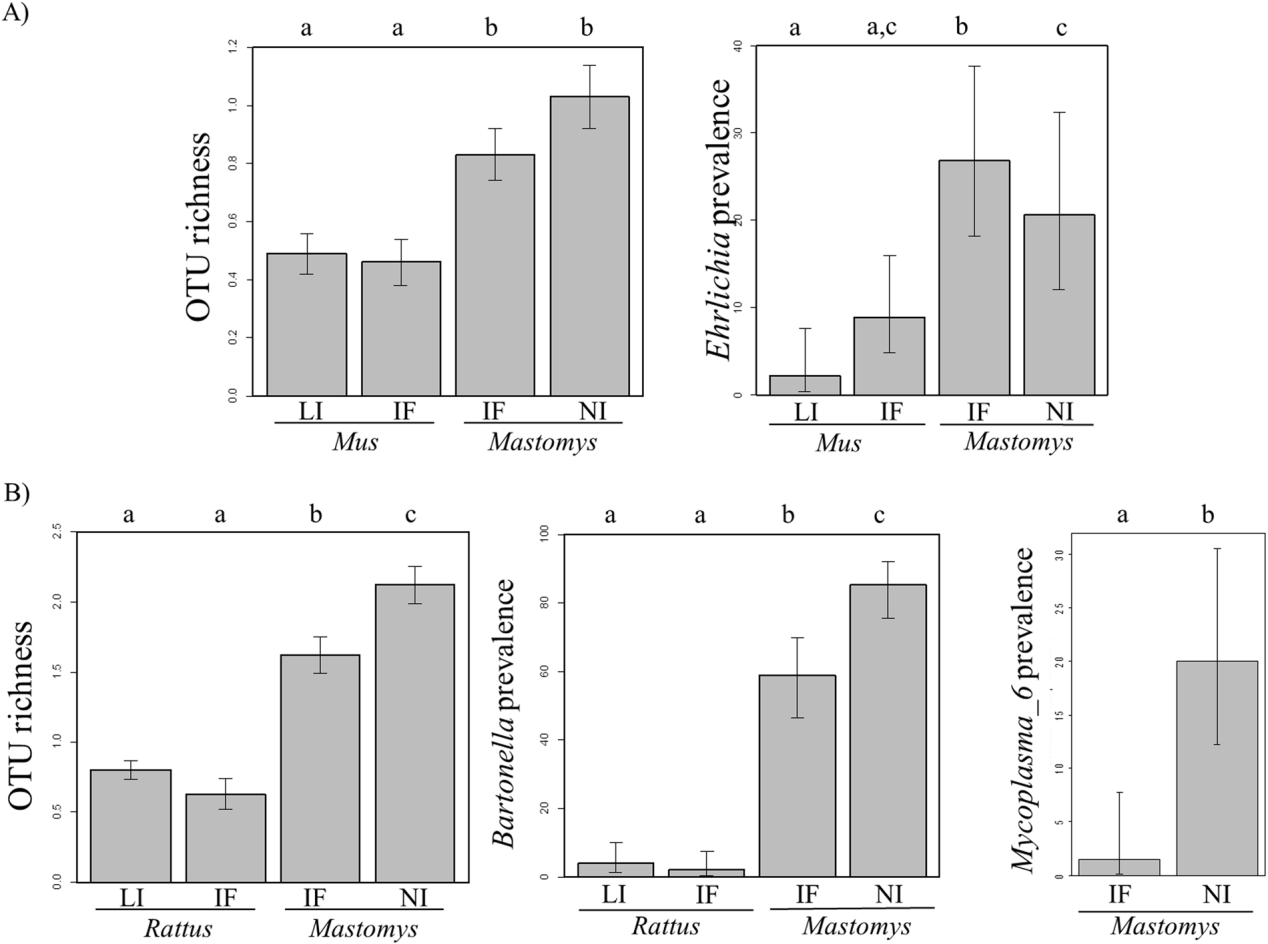
Significant differences in OTU prevalence and OTU richness (number of bacterial OTUs found in one host individual) between specific invasion-related categories of sites along the mouse (**A**) and rat (**B**) invasion routes. Only OTUs with an overall prevalence >5% in the dataset were analyzed. Error bars represent the standard error for richness data, and 95% confidence intervals calculated with Sterne’s exact method for prevalence data. Different letters above barplots indicate significant differences between two specific invasion-related categories. Legend: *Mus*: *Mus musculus domesticus*; *Mastomys*: *Mastomys erythroleucus* for the mouse invasion route, *Ma*. *erythroleucus* and *Mastomys natalensis* for the rat invasion route; *Rattus*: *Rattus rattus*; LI: sites of long-established invasion; IF: invasion front; NI: non-invaded sites.

For the mouse invasion route, generalised linear mixed models (GLMMs) (Fig. 4A; Supplementary Table S2) revealed significantly fewer bacterial OTUs in *M*. *m*. *domesticus* than in *Ma*. *erythroleucus* (*p* = *0*.*024*; pairwise Wilcoxon tests with Holm’s correction (WH tests), *p* < *0*.*05*). There was no difference in OTU richness between *M*. *m*. *domesticus* from the invasion front and from sites of long-established invasion (WH test, *p* = *0*.*99*), or between *Ma*. *erythroleucus* from the invasion front and from non-invaded sites (WH test, *p* = *0*.*39*). OTU richness was higher in males than in females (*p* = *0*.*001* for *sex* effect).

For the rat invasion route (Fig. 4B; Supplementary Table S2), invasive *R*. *rattus* rodents had fewer bacterial OTUs than native *Mastomys* (WH tests, *p* < *0*.*05*), and *Mastomys* individuals had fewer bacterial OTUs at the invasion front than at non-invaded sites (WH test, *p* = *0*.*008*). No significant difference was detected between *R*. *rattus* populations from the invasion front and from sites of long-established invasion (WH test, *p* = *0*.*580*). Finally, male and heavier rodents harboured more OTUs than female and lighter rodents (*p* < *0*.*001* for *sex* and *body mass* effects).

#### Bacterial OTU prevalence

Along the mouse invasion route (Fig. 4A; Supplementary Table S2), only *Ehrlichia* (*p* < *0*.*001*) displays a variation of infection levels that were higher in *Ma*. *erythroleucus* at the invasion front (WH tests, *p* < *0*.*005)*. Males were more frequently infected with *Mycoplasma_1* (*p* < *0*.*001*), *Ehrlichia* (*p* = *0*.*011*) and *Borrelia* (*p* = *0*.*002*) than females. For *Mycoplasma*_3, males more frequently infected at non-invaded sites than at the invasion front, and the opposite pattern was observed for females (*p* = *0*.*014*). Finally, heavier rodents were less frequently infected with *Ehrlichia* than lighter ones (*p* = *0*.*039*).

Along the rat invasion route (Fig. 4B; Supplementary Table S2), the infection rates of *Bartonella* (*p* < *0*.*001*) and *Mycoplasma*_6 (*p* = *0*.*006*) significantly vary. *R*. *rattus* was less frequently infected with *Bartonella* than *Mastomys* spp., even at the invasion front where they co-occurred (WH test, *p* < *0*.*001*). *Mastomys* spp. was also less frequently infected with *Bartonella* (WH test, *p* = *0*.*001*) and *Mycoplasma_6* (WH test, *p* = *0*.*004*) at the invasion front than at non-invaded sites. Males were more frequently infected with *Bartonella* (*p* = *0*.*025*), *Mycoplasma_1* (*p* = *0*.*004*), *Mycoplasma_4* (*p* = *0*.*02*) and *Mycoplasma_6* (*p* = *0*.*003*) than females. Heavier rodents were more frequently infected with *Mycoplasma 1* (*p* < *0*.*001*), *Mycoplasma_2* (*p* = *0*.*001*), *Mycoplasma 4* (*p* = *0*.*003*) and *Mycoplasma_5* (*p* = *0*.*036*) than lighter ones.

## Discussion

In this study, we investigated the variations of zoonotic bacterial communities in invasive and native rodents sampled along two invasion routes in Senegal. We performed high-throughput sequencing of 16S rRNA gene amplicon, an approach that has been commonly used these last years to characterize microbial communities and assess their diversity^17^. It has proven its strength when analysing large number of samples although some limitations with regards to taxonomical resolution due to the short read lengths obtained have been emphasized (e.g. Jovel *et al*.^19^; Poretsky *et al*.^20^). Despite this weakness, the application of 16S rRNA gene amplicon sequencing in a tropical context is an essential pre-requisite as there is still very few information concerning zoonotic microbial communities in wildlife and humans. This approach was therefore the first step to emphasize here (i) the factors underlying zoonotic bacterial community structure, (ii) the ecological hypotheses relating parasitism to invasion outcome in rodents, and (iii) the putative relationships between biodiversity and disease risks.

We found that host individual factors, such as body mass and sex, were important drivers of bacterial infections in rodents. Consistently with previous studies investigating relationships between mammalian hosts and their parasites^21,22^, we found that larger rodents and males were significantly more frequently infected than lighter rodents and females. Male-biased parasitism in rodents may result from profound sexual differences in anatomy, physiology, behaviour, and evolutionary roles, potentially increasing the likelihood of both encountering and acquiring parasites (reviewed in Krasnov *et al*.^23^). Larger individuals may have larger home ranges, increasing their frequency of contact with parasites^24^. Furthermore, as body mass may be considered to be a proxy for host age, the positive correlation generally found between infection and body mass may reflect the longer exposure times in older rodents. The only exception to this general pattern was *Ehrlichia* infection in *M*. *m*. *domesticus* and *Ma*. *erythroleucus*. This result suggested that older individuals would be less susceptible to infection, or that infected individuals would die rapidly.

Establishing the origin of parasites (native or exotic) is a key element when investigating hypotheses relating parasitism to invasion success. Assuming that a given OTU corresponded to a single bacterial taxa, the distribution of bacterial OTUs between native and invasive rodents may provide useful insight into this aspect. We found that some OTUs are probably native to the study area. Indeed, three OTUs (*Mycoplasma_3*, *Mycoplasma*_5, *and Mycoplasma*_6) were found mostly in *Mastomys* rodents. This may also be the case for *Ehrlichia*, which was detected at high prevalence in native rodents, but was found in invasive rodents almost exclusively at invasion fronts. The *Borrelia* OTU found in native and invasive rodents is also probably native, as large surveys have detected only one local species (e.g., *Borrelia crocidurae*) in the small mammal communities of West Africa^25,26^. Conversely, we can assume that four OTUs are exotic along both invasion routes. Two OTUs (*Mycoplasma_*2, *Mycoplasma_4*) along the rat invasion route were found repeatedly and exclusively in invasive *R*. *rattus*. The distribution of these bacteria suggests that they were introduced into Senegal along with this rodent. The detection of *Streptobacillus* in *R*. *rattus* at long-established invasion sites and in native rodents only at the invasion front of *R*. *rattus* also suggests an exotic origin for this OTU. The results concerning *Orientia* are a little bit more complex. In a previous study, we suggested an exotic origin for this genus^27^, which is found exclusively in *M*. *m*. *domesticus* along the mouse invasion route. However, the detection of sequences identical to those from this OTU in some native *Ma*. *natalensis* trapped outside the range of *M*. *m*. *domesticus* (Eastern Senegal) calls this hypothesis into question. The possibility that host species other than exotic rodents have played a role in the introduction of *Orientia* should also be considered (e.g., migratory birds^28^). Gathering molecular information to determine which *Orientia* strains circulate in native and exotic rodent species as well as birds would help deciphering these hypotheses about their origin in Senegal.

Besides, our results showed that bacterial communities varied along invasion routes and differed between invasive and native rodents. One key finding was that overall OTU richness was higher in native rodents than in invasive rodents, even when considering invasion fronts only. The absence of a signature of bacterial loss along invasion routes, regardless of the invasive species considered, does not necessarily invalidate the enemy release hypothesis, as these bacteria may have been lost at the time of *M*. *m*. *domesticus* and *R*. *rattus* introduction in Senegal. The mode of transmission and/or vector specificity of the introduced bacteria may also have favoured their persistence along the mouse and rat invasion routes. All the bacterial OTUs studied here, except *Mycoplasma* and *Streptobacillus*, have arthropod vectors (e.g., fleas, ticks, see Galan *et al*.^17^ and Ehounoud *et al*.^29^ for syntheses) that are highly likely to be present within the human dwellings sampled. For instance, the trombiculid mites involved in the transmission of *Orientia* ^27^ are known to have a very large host spectrum and low host specificity^28^, and these characteristics may have facilitated the introduction and/or persistence of this bacterium in new environments. The direct cycle of non-vectored bacteria, such as *Mycoplasma*, may also have enhanced their likelihood of establishment.

Different ecological processes connecting parasitism and invasion success received some support from the data gathered on native rodents, although we have to be cautious with these interpretations as they assumed that for a given OTU, the same bacterial strains circulate in native and exotic rodents. The higher prevalence of *Ehrlichia* in native rodents at the mouse invasion front compared with non-invaded sites could correspond to the expected pattern under the spill-back hypothesis. The infection of native *Mastomys* spp. by *Streptobacillus* at the invasion front of *R*. *rattus* could be the signature of a novel weapon process. Indeed, the sequence of *Streptobacillus* OTU exhibited 100% of identity with *Streptobacillus moniliformis* ^17^, a species that is known to be common and commensal in rats, but pathogenic in other rodents^30^. Finally, the lower prevalence of *Bartonella* and *Mycoplasma_6* prevalence in native rodents at the invasion front than at non-invaded sites along the rat invasion route could correspond to the pattern expected under the dilution/density hypotheses. Going further in the examination of ecological processes connecting parasitism and invasion success would require the evaluation of bacteria pathogenicity in native and invasive rodents through experimental studies, and a finer identification of bacterial OTUs at the species or even strain level.

We faced the same limitations in taxonomic resolution when analysing the potential epidemiological implications of this study. Nevertheless, the 251 bp 16S rRNA fragment sequenced in this study provided taxonomic identification down to the genus level in most cases^17^. Our results are therefore of main importance in this geographical context as epidemiological research on zoonotic bacteria infecting wild rodents in continental Africa remains scarce. Most studies have focused on *Yersinia pestis* ^31^, *Borrelia* sp.^25^, *Bartonella* sp.^32^, and *Leptospira* sp.^33^. This study provides information about the geographic distribution of four other bacterial genera (*Ehrlichia*, *Orientia*, *Rickettsia*, *Streptobacillus*) with some species being zoonotic agents capable of causing severe human diseases in Africa, and which remain underdiagnosed (e.g. Ehounoud *et al*.^29^). For instance, extensive surveys of zoonotic *Borrelia* in West African rodents (e.g., Trape *et al*.^25^ and references therein) provided evidence that the *Borrelia* OTU was from *B*. *crocidurae* (100% of percentage identity on the 251 bp studied^17^), the only species identified in various rodent species in Senegal, known to cause severe borreliosis in humans^25^. The *Orientia* genus numbers only two species that are both known to be zoonotic, and the *Orientia* OTU found here could be *O*. *chuto* (100% of percentage identity^27^). Finally, the sequences of the six *Mycoplasma* OTUs had all high percentages of identity with species known to be zoonotic^17^. Besides, if we confirmed that the same bacterial strains circulate in native and exotic rodents, our results would suggest that invasive rodents may have transported exotic bacteria to the invasion fronts (e.g. *Streptobacillus* or *Rickettsia* in South-Eastern Senegal, transported by *R*. *rattus*), may affect the prevalence of some of the bacteria carried by native rodents (e.g., increase in *Ehrlichia* and decrease in *Bartonella* levels), or may lead to the replacement of local lineages or species of *Mycoplasma* with exotic ones. We highlighted that both qualitative and quantitative OTU composition varied between the three invasion-related categories of sites sampled irrespective of the invasion route considered (Fig. 2). All these patterns suggested that vigilance should be increased along the invasion routes of exotic rodents to monitor changes in the risks of rodent-borne zoonotic diseases.

In conclusion, this study on rodent invasion and parasitism supported the hypothesis that the relationships between biodiversity and disease risk cannot be easily predicted^34^, particularly when tracking multiple diseases^35^. A next crucial step should be to identify these bacterial genera to the species level using specific molecular assays, in order to decipher concretely their ecological and epidemiological implications. We would argue that public health prevention strategies based on multiple pathogen surveillance are required, especially for invasive reservoir/vector species monitoring^36^. This is even more true in tropical regions, in which many infectious diseases originating from wildlife are still underreported.

## Methods

### Sample collection

We used data from historical records and population genetics studies^9–11^; together with data from longitudinal sampling surveys of rodent communities in Senegal carried out since the 1980s (http://www.bdrss.ird.fr/bdrsspub_form.php ^12^) to select sampling sites (villages or towns) and assign them to three categories for each invasion route: (i) coastal sites of long-established invasion, where rats/mice have settled in large and permanent populations since at least the 18^th^–9^th^ century and have excluded native rodents, (ii) sites at the invasion front, at which invasive rodents arrived only recently (10–30 years ago) and currently coexist with native rodents, and (iii) non-invaded sites where only native species are known to occur. Three to six sites were systematically sampled for each category (Fig. 1), outside the area of sympatry of both invasive rodent species to avoid any confounding effects. Details on rodent trapping and identification, autopsy procedures and age determination are provided elsewhere^9,15^. For bacterial 16S gene rRNA amplicon sequencing, spleens were carefully removed mostly from adult rodents except when sample size was too small in a site. They were placed in RNAlater storage solution, stored at 4 °C overnight and then at −20 °C until further analyses.

### Bacterial 16S gene rRNA amplicon sequencing

Data for the bacterial communities considered here were obtained with a 16S gene rRNA amplicon sequencing approach, as previously described^17^. This approach provided an almost complete inventory of the bacterial community infecting the rodents sampled. Briefly, genomic DNA was extracted from the spleen with the DNeasy 96 Tissue Kit (Qiagen) and subjected to PCR amplification with slightly modified versions of the universal primers used in a previous study (Kozich, *et al*. 2013) to amplify a 251 bp portion of the 16S rRNA V4 region (V4F: GTGCCAGCMGCCGCGGTAA; V4R: GGACTACHVGGGTWTCTAATCC). The amplicons were then sequenced on an Illumina MiSeq sequencer. The hypervariable region of the 16S rRNA gene studied here can be used for the accurate taxonomic assignment of diverse bacteria, at the genus level and even below in some cases^37^. The technical procedures and bioinformatics analyses used here have been presented in detail elsewhere^17^. Briefly, the MiSeq data were processed with MOTHUR v1.34 sofware, and taxonomic assignment was performed with the SILVA SSU Ref database v119 as a reference and refined by phylogenetic analyses and BLAST analyses against GenBank data. The reads generated by Illumina MiSeq sequencing were clustered into operational taxonomic units (OTUs) with a 3% divergence threshold. Finally, the data were filtered to eliminate false-positive and false-negative results for each bacterial OTU. The presence of a given OTU within a given rodent was then validated with systematic technical replicates. The results were then summarised in a presence/absence dataset, with only bacterial OTUs known or suspected to be zoonotic retained for subsequent analyses (see Galan *et al*. for details^17^).

### Data analyses

Each invasion route was investigated separately.

We investigated whether the composition of these zoonotic bacterial communities was structured at site level by host species and/or the invasion-related category of sites at intraspecific level, by constructing dissimilarity matrices based on the Bray-Curtis (BC) β-diversity index from data counts for infected individuals in each host population. The structure of the bacterial communities was then visualised with a hierarchical clustering approach based on BC dissimilarity matrices, using a Ward linkage function^38^. The effects of host species and specific invasion-related category were assessed in a permutational multivariate analysis of variance (permanova) on distance matrices. These analyses were carried out with the R packages Vegan v2.4.2^39^ and Phyloseq v1.19.1^40^.

We then used individual-level generalised linear mixed models (GLMMs) to test for patterns consistent with the enemy release, novel weapon, spillback, and dilution/density hypotheses. We used individual bacterial richness (number of OTUs recorded in a single individual host) and OTU prevalence (infection with a specific OTU for which prevalence in the dataset reached at least 5%) as response variables. For OTUs found exclusively or mainly in a single host species (more than 95% of infected hosts), variations of specific prevalence were investigated with a dataset restricted to individuals of this host species only. We assumed a binomial distribution (quasibinomial in cases of overdispersion) for incidence data and a Poisson distribution (negative binomial in cases of overdispersion) for bacterial richness data. We prevented confounding effects by considering a single factor ‘*specific invasion-related category*’, which combined both the host species and the invasion-related category of the sampling site along the invasion route. Four categories were defined for this factor: (A) invasive species at a site of long-established invasion; (B) invasive species at the invasion front; (C) native species at the invasion front and (D) native species at a non-invaded site. The full model included individual host factors (*sex* and *body mass*), *specific invasion-related category* (A, B, C and D) and their pairwise interactions. We considered *sampling site* as a random factor. The best-fitting model was selected on the basis of a goodness-of-fit criterion, Akaike’s information criterion, with correction for samples of finite size (AICc). We then chose the most parsimonious model from those selected within two AIC units of the best model. *P*-values were obtained by stepwise model simplification in likelihood-ratio tests^41^. For each final model, linear regression residuals were checked to ensure that regression assumptions regarding normality, independence and the homogeneity of variance were satisfied^41^. Multiple pairwise comparisons were performed with Wilcoxon tests and Holm’s correction method for *p-*value adjustment. All model analyses were performed with the R packages lme4 v1.1–8^42^ and MuMIn v1.15.1^43^.

### Ethics statements

Trapping campaigns within villages and on private land were conducted with the authorisation of the appropriate institutional and familial authorities. None of the rodent species investigated here has protected status (see list of the International Union for Conservation of Nature). All procedures and methods were carried out in accordance with relevant regulations and official guidelines from the American Society of Mammalogists^44^. All protocols presented here were realized with prior explicit agreement from relevant institutional committee (Centre de Biologie pour la Gestion des Populations (CBGP): 34 169 003). The spleen samples were sent in dry ice to Montpellier (France) and were stored in an experimental animal room at the CBGP. (34-169-1). All transfer and conservation procedures were performed in accordance with current international legislation.

### Data availability

Data for this study will be made available via Dryad (DataDryad.Org) once the manuscript has been accepted for publication.

## Electronic supplementary material

Supplementary Tables S1 & S2
