# Supplementary material for: Ecological and sanitary impacts of bacterial communities associated to biological invasions in African commensal rodent communities
Source: Sci Rep. 2017 Nov 3;7:14995. doi: 10.1038/s41598-017-14880-1 (PMC5670214; doi:10.1038/s41598-017-14880-1)
Supplement: Supplementary file 1 — Supplementary Tables S1 & S2 [file 41598_2017_14880_MOESM1_ESM.docx]

**Ecological and sanitary impacts of bacterial communities associated to biological invasions in African commensal rodent communities.**

Christophe Diagne^1,2,3*^, Maxime Galan^4^, Lucie Tamisier^4^, Jonathan d’Ambrosio^4^, Ambroise Dalecky^5^, Khalilou Bâ^2^, Mamadou Kane^2^, Youssoupha Niang^2^, Mamoudou Diallo^2^, Aliou Sow^2^, Philippe Gauthier^1^, Caroline Tatard^4^, Anne Loiseau^4^, Sylvain Piry^4^, Mbacké Sembène^2,3^, Jean-François Cosson^6^, Nathalie Charbonnel^4^ & Carine Brouat^1^

^1^ CBGP, IRD, CIRAD, INRA, Montpellier SupAgro, Univ. Montpellier, Montpellier, France

^2^ IRD, CBGP (UMR INRA / IRD / Cirad / Montpellier SupAgro**)**, Campus ISRA/IRD de Bel Air, Dakar, Senegal

^3^ Département de Biologie Animale, Faculté des Sciences et Techniques, Université Cheikh Anta Diop (UCAD), BP 5005 Fann, Dakar, Senegal

^4^ CBGP, INRA, CIRAD, IRD, Montpellier SupAgro, Univ. Montpellier, Montpellier, France.

^5^ IRD, Aix Marseille Univ, LPED, Marseille, France.

^6^ INRA, Anses, USC Bipar, Bartonella et Tiques, 23 Avenue du Général de Gaulle, 94700 Maisons-Alfort, France

Corresponding author: Christophe Diagne. E-mail: chrisdiagne89@hotmail.fr

**Supplementary Table S1**: Sampling sites and their invasion-related category as well as rodent sample size per site on A) the mouse invasion route and B) the rat invasion route. Legend: LI: sites of long-established invasion; IF: recently invaded sites (invasion front); NI: non-invaded sites; Lat: latitude of sites; Long: longitude of sites; N: total number of rodents sampled (M: males; F: females). ‘-’ indicates no data (no rodent sampled or screened).

A)

| **Sites (Invasion related category)** | **Lat (North)** | **Long (West)** | ***Mus musculus domesticus*** | ***Mastomys erythroleucus*** |
| --- | --- | --- | --- | --- |
|  |  |  | **N (M/F)** | **N (M/F)** |
| Dagathie (LI) | 15.62 | -16.25 | 30 (18/12) | - |
| Mbakhana (LI) | 16.08 | -16.36 | 30 (14/16) | - |
| Ndombo (LI) | 16.43 | -15.70 | 23 (10/13) | - |
| Thilene (LI) | 16.26 | -16.18 | 26 (9/17) | - |
| Aere Lao (IF) | 16.40 | -14.32 | 50 (22/28) | 22 (13/9) |
| Croisement Boube (IF) | 16.49 | -15.00 | 45 (24/21) | 3 (2/1) |
| Dendoudi (IF) | 15.39 | -13.53 | 19 (13/5) | 28 (18/10) |
| Dodel (IF) | 16.48 | -14.42 | 24 (9/15) | 33 (16/17) |
| Lougue (IF) | 16.06 | -13.91 | 28 (13/15) | 25 (15/10) |
| Diomandiou Walo (NI) | 16.49 | -15.00 | - | 14 (10/4) |
| Doumnga Lao (NI) | 16.33 | -14.22 | - | 43 (18/25) |
| Lambango (NI) | 15.46 | -13.53 | - | 23 (10/13) |
| Sare Maounde (NI) | 15.98 | -13.91 | - | 9 (5/4) |

B)

| **Sites (Invasion related category)** | **Lat (North)** | **Long (West)** | ***Rattus rattus*** | ***Mastomys erythroleucus*** | ***Mastomys natalensis*** |
| --- | --- | --- | --- | --- | --- |
|  |  |  | **N (M/F)** | **N (M/F)** | **N (M/F)** |
| Diakene-Wolof (LI) | 12.45 | -16.64 | 30 (12/18) | 8 (5/3) | - |
| Diattacounda (LI) | 12.57 | -15.68 | 35 (19/16) | 16 (9/7) | - |
| Marsassoum (LI) | 12.83 | -15.97 | 29 (13/16) | 3 (0/3) | - |
| Tobor (LI) | 12.66 | -16.25 | 29 (12/17) | 1 (1/0) | - |
| Badi Nieriko (IF) | 13.37 | -13.37 | 73 (35/38) | 14 (6/8) | - |
| Boutougoufara (IF) | 13.39 | -12.48 | 40 (15/25) | 21 (12/9) | - |
| Kedougou (IF) | 12.55 | -12.17 | 34 (13/21) | - | 30 (11/19) |
| Soutouta (IF) | 13.80 | -12.71 | 26 (13/13) | 12 (5/7) | - |
| Bransan (NI) | 13.26 | -12.10 | - | 6 (4/2) | 33 (14/19) |
| Mako (NI) | 12.85 | -12.35 | - | - | 48 (28/20) |
| Segou (NI) | 12.41 | -12.28 | - | - | 26 (10/16) |

**Supplementary Table S2:** Factors shaping bacterial OTU richness and OTU prevalence evidenced by generalized linear mixed models along both invasion routes. Only OTUs with an overall prevalence > 5% in the dataset considered were analysed. Variations in OTU prevalence were tested using a dataset restricted to a single host species when at least 95% of infected hosts by an OTU were from one species. Only models having significant explanative factors and validated by residual checking were presented. Significant factors are those from the most parsimonious generalized linear mixed model obtained after model selection and residuals-based validation procedures carried out for each response variable. AICc: Akaike’s information criterion with correction for finite sample size. Δ indicates the difference between the model selected and the model with the lowest AICc. Sex: males *vs* females. LRT: Likelihood-ratio test value. *P-value* was considered significant when < 0.05. “A:B” means interaction between factor A and factor B.

| Invasion route | Response variable |  | Dataset considered |  | AICc (Δ) | Significant factors |  | Df | LRT | *p-value* |
| --- | --- | --- | --- | --- | --- | --- | --- | --- | --- | --- |
| Mouse | *Borrelia* |  | whole |  | 165.8 (1.95) | sex |  | 1 | 10.04 | 0.002 |
|  | *Ehrlichia* |  | whole |  | 241.3 (0.00) | body mass |  | 1 | 4.27 | 0.039 |
|  |  |  |  |  |  | sex |  | 1 | 6.54 | 0.011 |
|  |  |  |  |  |  | specific invasion-related category |  | 3 | 7.46 | < 0.001 |
|  | *Mycoplasma 1* |  | whole |  | 267.0 (1.24) | sex |  | 1 | 48.57 | < 0.001 |
|  | *Mycoplasma 3* |  | only natives |  | 160.2 (0.14) | sex : invasion-related category |  | 3 | 6.1 | 0.014 |
|  | OTU richness |  | whole |  | 701.9 (0.53) | sex |  | 1 | 11.32 | 0.001 |
|  |  |  |  |  |  | specific invasion related category |  | 3 | 9.41 | 0.024 |
| Rat | *Bartonella* |  | whole |  | 202.7 (0.08) | sex |  | 1 | 5.03 | 0.025 |
|  |  |  |  |  |  | specific invasion-related category |  | 3 | 54.84 | < 0.001 |
|  | *Mycoplasma 1* |  | only natives |  | 148.6 (0.82) | body mass |  | 1 | 22.6 | < 0.001 |
|  |  |  |  |  |  | Sex |  | 1 | 22.88 | 0.004 |
|  | *Mycoplasma 2* |  | whole |  | 219.0 (0.00) | body mass |  | 1 | 11 | 0.001 |
|  | *Mycoplasma 4* |  | whole |  | 92.6 (0.00) | body mass |  | 1 | 8.98 | 0.003 |
|  |  |  |  |  |  | sex |  | 1 | 5.39 | 0.02 |
|  | *Mycoplasma 5* |  | only natives |  | 79.3 (0.00) | body mass |  | 1 | 4.42 | 0.036 |
|  | *Mycoplasma 6* |  | only natives |  | 82.3 (0.00) | sex |  | 1 | 8.9 | 0.003 |
|  |  |  |  |  |  | specific invasion-related category |  | 1 | 7.6 | 0.006 |
|  | OTU richness |  | whole |  | 825.4 (0.00) | body mass |  | 1 | 20.47 | < 0.001 |
|  |  |  |  |  |  | sex |  | 1 | 14.46 | < 0.001 |
|  |  |  |  |  |  | specific invasion-related category |  | 3 | 62.99 | < 0.001 |
